# Temporal Feature Interaction for Robust Remote Sensing Image Change Detection: A Taxonomy and Cross-Domain Comparative Study

**DOI:** 10.3390/jimaging12080376

**Published:** 2026-08-11

**Authors:** Mostafa Mosaad, Mahmoud Ahmed, Fawzy Eltohamy, Tarek A. Mahmoud, Mohamed E. Hanafy

**Affiliations:** 1 Department of Aircraft Electrical Equipment, Military Technical College, Cairo 11766, Egypt; ftohamy72@yahoo.com (F.E.); mehanafy@mtu.edu (M.E.H.); 2 Department of Electrical and Software Engineering, University of Calgary, Calgary, AB T2N 1N4, Canada; 3 Computer Engineering Department, Egypt University of Informatics, Cairo 19519, Egypt; tarek.mohamed@eui.edu.eg

**Keywords:** remote sensing, change detection, deep learning, bi-temporal fusion, transformers, cross-dataset generalization, Open-CD

## Abstract

Remote sensing change detection (RSCD) has advanced through convolutional, attention-based, transformer, and hybrid architectures, yet models are commonly compared as whole architectural families rather than by how their temporal streams interact. This study introduces the Temporal Interaction Taxonomy (TIT), which characterizes temporal feature interaction by timing, direction, and operator. Nine representative models, ranging from early-fusion convolutional baselines to hybrid CNN–Transformer designs, were evaluated using faithful Open-CD implementations under a unified protocol on the LEVIR-CD and WHU-CD building change datasets. TIT provided a consistent basis for describing bi-temporal integration across architectures. Cross-dataset performance was model- and direction-dependent: ChangeFormer achieved the highest mIoU in both transfer directions, reaching 59.99% for WHU→LEVIR and 82.58% for LEVIR→WHU. The results suggest an association between interaction design and cross-dataset robustness, but the independent contribution of temporal interaction cannot be separated from other architectural differences. Transfer also showed model-dependent directional asymmetry; its causes could not be isolated because dataset characteristics and model design were not independently controlled. Overall, temporal interaction provides a useful dimension for interpreting model behavior across the two evaluated datasets. These findings are limited to building change detection on LEVIR-CD and WHU-CD and require task-specific validation before extension to other RSCD applications.

## 1. Introduction

Change detection (CD) in remote sensing imagery underpins applications such as urban expansion monitoring, disaster assessment, and post-event reconstruction [1]. Given two co-registered images, $I_{t_{1}}$ and $I_{t_{2}},$ RSCD produces a pixel-wise change map. Deep learning now dominates the field for its hierarchical representations [2], though very-high-resolution imagery remains challenging: illumination, seasonal, and sensor artifacts can mimic change, while subtle real changes are easily missed [3]. Across RSCD architectures, one operation recurs: how information from $I_{t_{1}}$ and $I_{t_{2}}$ is combined. Early approaches stack the images as multi-channel input; dual-stream designs extract features separately and fuse them later via concatenation or differencing [4]; and recent models add attention-guided exchange, token-based aggregation, or explicit inter-stream communication [5,6,7,8]. Yet existing research still analyzes RSCD mainly at the level of overall architecture (CNN, Transformer, hybrid), treating interaction as an implementation detail rather than a unified analytical dimension.

Instead of comparing architectures alone, this study focuses on TFI as the key analytical dimension for understanding RSCD behavior under domain shift, with three main contributions:

- Temporal Interaction Framework: A unified framework for how bi-temporal information is integrated in RSCD models, structured along three orthogonal axes, interaction timing, direction, and operator, enabling a principled account of model behavior.
- Interaction-centric model interpretation: Representative RSCD models are grouped by interaction mechanism rather than broad architecture family, explaining performance differences, characteristic failure modes, and robustness behavior.
- Empirical evidence under domain shift: Nine widely used RSCD models, using official Open-CD implementations [9], are evaluated on LEVIR-CD [10] and WHU-CD [11] under both in-domain and cross-domain protocols, enabling interaction-oriented interpretation of the observed generalization patterns.

Together, these contributions position TFI as a relevant analytical dimension for interpreting robustness, region-overlap performance, and qualitative spatial patterns in RSCD. This interaction-oriented perspective complements conventional architectural classifications and echoes recent concerns that architectural novelty alone may not reflect real progress [2].

## 2. Background and Related Work

### 2.1. From Classical RSCD to Deep Dense Prediction

Early RSCD methods relied on handcrafted descriptors and post-processing techniques such as thresholding, which struggled under real-world appearance variability [2,3]. Deep learning reframed RSCD as a dense prediction task, with encoder–decoder designs (e.g., U-Net) enabling pixel-level change maps [12]. The now-standard dual-stream (Siamese) paradigm encodes $I_{t_{1}}$ and $I_{t_{2}}$ separately, often with shared weights, before fusing them [4]. This architecture cleanly separates representation learning from temporal fusion, which later became the main axis of architectural innovation.

### 2.2. Temporal Fusion as the Hidden Design Decision

Although RSCD models differ in macro-structure, their behavior often hinges on one core operation: how features from $I_{t_{1}}$ and $I_{t_{2}}$ are fused. Simple concatenation or differencing works well in-domain but frequently mistakes illumination shifts or misregistration artifacts for actual change [4]. Timing matters equally: early fusion combines images at the input level, while Siamese designs delay fusion until after independent feature extraction [4]. Although attention-based fusion later added selective feature emphasis, it did so at the cost of extra computation [2,3]. These observations motivate treating fusion as a first-class design choice rather than a minor implementation detail.

### 2.3. Interaction-Rich Designs: Attention, Tokens, and Exchange

Interaction-rich designs have since moved beyond single-shot fusion mechanisms. Transformer mechanisms model long-range spatial–temporal dependencies to yield structurally consistent change maps [6,7]. For instance, BIT enhances convolutional features with semantic tokens [6], whereas ChangeFormer computes multi-scale bi-temporal differences within a Siamese transformer architecture [7]. More recently, exchange-oriented designs explicitly pass information between streams to reduce feature ambiguity across time steps [8], and open-source toolboxes such as Open-CD have successfully standardized implementations for reproducible comparison [9].

### 2.4. Generalization and Domain Shift in RSCD

A persistent RSCD challenge is that strong benchmark scores do not reliably translate to real-world deployment. Many datasets are collected under consistent acquisition conditions that reward models for learning dataset-specific appearance cues rather than transferable, invariant notions of change [2,3]. Distribution shifts triggered by new cities, seasons, or sensor modalities cause models to over-predict change in stable areas or miss subtle structural changes [2,3]. These characteristic failure modes—specifically appearance change confusion, boundary fragmentation, and small object omission—are tightly linked to how temporal information is combined, motivating an analytical lens centered on interaction rather than broad model categories.

Recent adaptation-oriented RSCD studies have addressed domain shift through several complementary mechanisms. CSDACD, a domain-adaptive change detection network designed for cross-seasonal remote sensing imagery, integrates domain-adapted images into an end-to-end change detection network and employs semantic change alignment [13]. IRD-CD-UDA, an illumination–reflection decoupling-based change detection model with unsupervised domain adaptation, combines illumination–reflection feature decoupling with multiscale fusion and cross-domain feature alignment [14]. For heterogeneous remote sensing imagery, domain-adaptive cross-reconstruction uses feedback guidance to align information across imaging modalities [15]. Test-time adaptation (TTA) represents a related but distinct deployment strategy in which a pretrained model is updated at test time using unlabeled target-domain samples, for example through entropy minimization or self-training [16,17]. Unlike these approaches, which seek to improve target-domain performance by modifying the training or inference procedure, the present study deliberately adopts a source-only protocol: each model is trained on the source dataset and evaluated directly on the target dataset without using target-domain samples for training or model adaptation. This controlled cross-dataset setting therefore evaluates the transfer behavior of the original architectures rather than their ability to adapt once target-domain samples become available.

### 2.5. An Interaction-Centric Perspective on RSCD Models

This work differs from conventional benchmarking frameworks in two distinct ways. First, we organize existing models around TFI axes—specifically timing, direction, and operator—to examine how their interaction profiles relate to behavior under appearance-driven variability [4,5,6,7,8]. Second, our experiments rely on reproducible official Open-CD implementations [9] to compare the observed behavior of these models under both in-domain and cross-domain protocols. This approach provides an interaction-oriented account of the observed strengths and failure patterns and supports context-dependent model interpretation under the evaluated conditions.

Conventional fusion taxonomies classify bi-temporal integration according to where it occurs, typically distinguishing early, intermediate, and late fusion. TIT retains this stage-based perspective through its timing axis, while adding two further dimensions: the direction of information flow and the interaction operator. It therefore complements, rather than replaces, existing fusion taxonomies. TIT and adaptation methods likewise address different aspects of a change-detection system. TIT characterizes how bi-temporal information interacts within the architecture, whereas domain adaptation and test-time adaptation determine how the model is trained or updated when transferred across domains. In principle, an adaptation strategy can be applied to any TIT profile and may operate before, within, or after the temporal interaction module. The present study neither compares adaptation strategies nor suggests that TIT subsumes them; rather, TIT is used solely to interpret unchanged models evaluated under a common source-only transfer protocol.

## 3. Temporal Interaction Taxonomy (TIT) for Model Analysis

**Figure 1:**
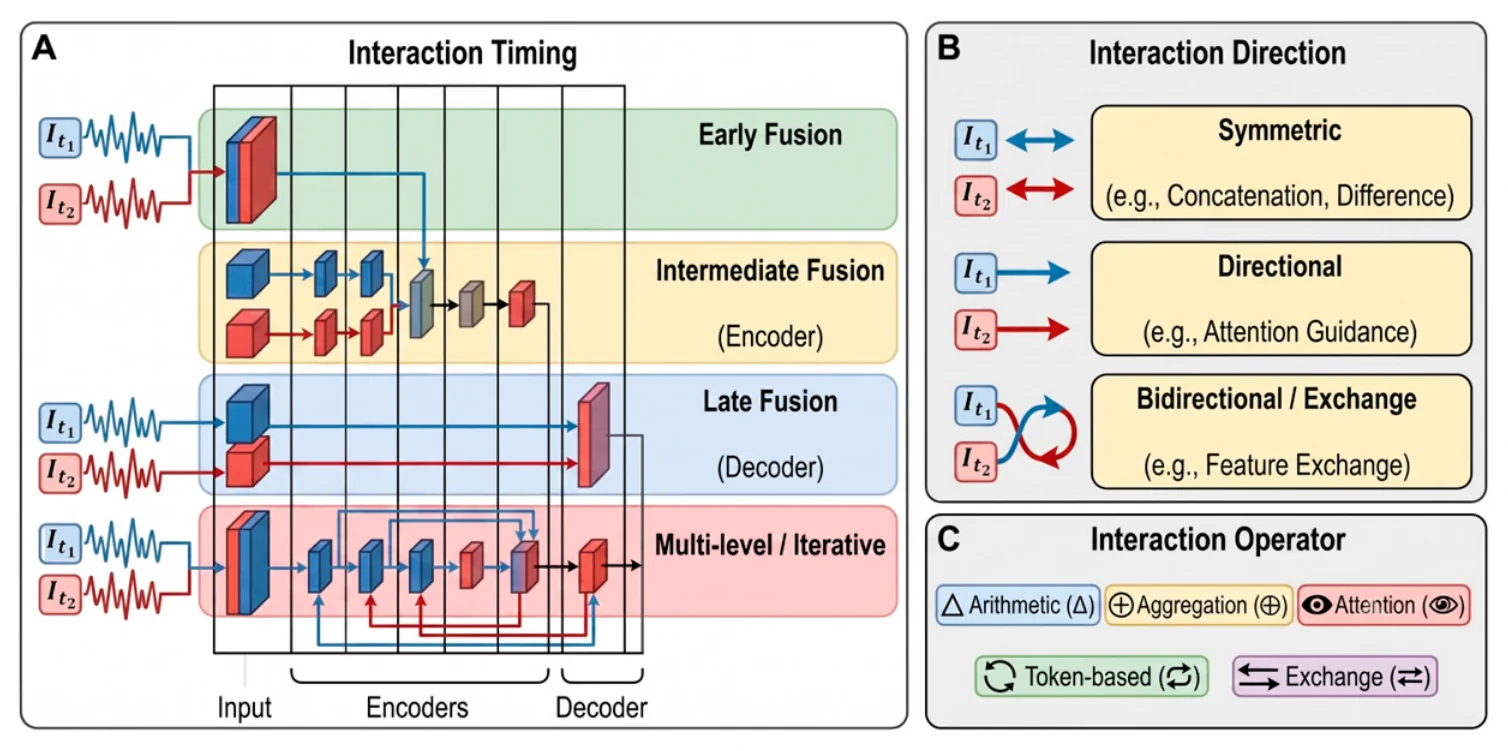
Temporal Interaction Taxonomy for Change Detection. (**A**) Interaction timing: early, intermediate (encoder), late (decoder), and multi-level or iterative fusion between $I_{t_{1}}$ and $I_{t_{2}}$ (**B**) Interaction direction: symmetric, directional, and bidirectional or exchange patterns. (**C**) Interaction operator: arithmetic, aggregation, attention, token-based, and explicit exchange mechanisms. Blue and red denote the feature streams corresponding to the two temporal inputs, while arrows indicate the direction of information flow.

Temporal interaction is the process by which features derived from $I_{t_{1}}$ and $I_{t_{2}}$ are combined, compared, or progressively refined to support change prediction. Proposed designs can be summarized along three axes: interaction timing, interaction direction, and interaction operator. As shown in Figure 1, these axes provide a compact framework for describing how temporal information is integrated within RSCD models.

In this setting, the process begins with an ordered bi-temporal input pair comprising two co-registered images acquired at times $t_{1}$ and $t_{2}$:

(1) $$X=(I_{t_{1}},I_{t_{2}})$$

Here, $I_{t_{1}}$ and $I_{t_{2}}$ denote the co-registered images acquired at times $t_{1}$ and $t_{2}$, respectively, and X denotes the corresponding ordered bi-temporal input pair. Rather than comparing these images only at the pixel level, an encoder (*E*) first maps each image into a hierarchy of feature representations. At network level (*l*), this can be written as:

(2) $$F_{t_{1}}^{l}=E_{l}\left(I_{t_{1}}\right),\;\;\;\;\;\;\;\;\;F_{t_{2}}^{l}=E_{l}(I_{t_{2}})$$

Here, $E_{l}$ denotes the encoder mapping at network level *l*, while $F_{t_{1}}^{l}$ and $F_{t_{2}}^{l}$ denote the corresponding feature representations extracted from $I_{t_{1}}$ and $I_{t_{2}}$, respectively. The key design issue is how the two feature representations are allowed to interact. At network level *l*, this interaction is represented by the temporal interaction function $\Phi_{l}$:

(3) $$Z^{l}=\Phi_{l}(F_{t_{1}}^{l},F_{t_{2}}^{l};\tau ,d,o)$$

Here, $Z^{l}$ denotes the resulting temporally interacted feature representation at network level *l*, and $\Phi_{l}$ denotes the interaction function applied at that level. The taxonomy variables τ,d, and o represent the three dimensions of the proposed framework: τ specifies when the interaction occurs, d specifies the direction of information flow between the temporal streams, and o specifies the operator used to combine, compare, or refine the feature representations. Finally, the interaction representations obtained from one or more network levels are passed to a decoder or prediction head G to estimate the final change map:

(4) $$\hat{Y}=G(\{{Z^{l}\}}_{l=1}^{L})$$

Here, $\hat{Y}$ denotes the predicted change map, G denotes the decoder or prediction head, and L denotes the number of network levels whose interaction representations are provided to G. This formulation provides a concise mathematical description of the overall process: the bi-temporal image pair is first encoded into feature representations, the resulting temporal feature streams interact according to the timing, direction, and operator dimensions of the proposed taxonomy, and the interaction representations are subsequently decoded to produce the final change map.

### 3.1. Interaction Timing: When Are Features Integrated?

Interaction timing refers to the network stage at which the feature streams derived from $I_{t_{1}}$ and $I_{t_{2}}$ are integrated. Early fusion (input level) captures direct pixel differences but is noise-sensitive; intermediate fusion combines more abstract encoder features for robustness; late fusion (near the decoder) preserves temporal semantics but can weaken boundaries; and multi-level/iterative interaction spans several stages for progressive refinement.

### 3.2. Interaction Direction: How Information Flows Between $I_{t_{1}}$ and $I_{t_{2}}$

Interaction direction describes how information flows between $I_{t_{1}}$ and $I_{t_{2}}$: symmetric interaction treats both equally (concatenation, difference, correlation); directional interaction uses one to guide the other, risking directional bias; and bidirectional (exchange) interaction refines both representations jointly.

### 3.3. Interaction Operator: Mechanisms of Feature Interaction

Interaction operators define the integration mechanism: arithmetic (Δ, e.g., subtraction) highlights discrepancies; aggregation (⊕, e.g., concatenation) enables learned fusion; attention selectively weights temporal evidence; token-based (T) models relationships via compact tokens; and exchange (⇄) explicitly passes information between streams.

### 3.4. Taxonomic Mapping and Interaction-Centric Interpretation of Failure Modes

The proposed TIT serves as an interpretability framework: each model can be described by when interaction occurs, how information flows, and which operator is used. This interaction-centered lens helps explain common failure cases, including appearance-change confusion, boundary fragmentation, missed small objects, and weak cross-domain robustness.

## 4. Experimental Work (Datasets and Implementation)

Building change detection is emphasized here for its practical importance in urban monitoring, damage assessment, and reconstruction analysis, and its wide representation in RSCD benchmarks [2,3,10,11]. Two complementary very-high-resolution datasets are used for evaluation.

- LEVIR-CD [10]: A widely used benchmark for urban building change detection comprising 637 bi-temporal image pairs of 1024 × 1024 pixels collected from 20 geographically separate regions. Following the standard partition, 445 pairs were used for training, 64 for validation, and 128 for testing.
- WHU-CD [11]: A large-scale building change dataset collected in a post-earthquake reconstruction context. For the experiments in this study, the original geographically contiguous bi-temporal imagery was divided into 434 non-overlapping image pairs of 1024 × 1024 pixels, comprising 226 training pairs, 54 validation pairs, and 154 test pairs.

Thus, although the input dimensions were standardized to 1024 × 1024 pixels, the two datasets differed in the number of training pairs and in their geographical sampling structures. These characteristics provide complementary conditions for evaluating both in-domain performance and zero-shot cross-domain generalization.

### 4.1. RSCD Models in the Open-CD Framework

All models were evaluated using their official Open-CD configurations to preserve implementation fidelity and reproducibility [9], except that SyncBN layers were converted to standard BatchNorm to enable single-GPU execution. No other architectural components were modified. The evaluated models represent the interaction behaviors examined through TIT—including simple-fusion, attention-guided, token-based, and exchange mechanisms—without constituting an exhaustive survey. Their analytical assignments are summarized in Table 1.

For discussion purposes, the evaluated models are organized into three analytical, interaction-oriented categories derived from TIT; these categories are specific to the present analysis and are not proposed as a universal architectural classification:

- Category A—Simple-Fusion Baselines (Arithmetic/Aggregation): FCEF, FC-Siam-Conc, and FC-Siam-Diff [4].

FCEF is an early-fusion, single-stream model that stacks $I_{t_{1}}$ and $I_{t_{2}}$ at the input and therefore does not follow the classical Siamese dual-stream formulation. In contrast, FC-Siam-Conc and FC-Siam-Diff use Siamese-style dual streams with explicit fusion operators [4].

- Category B—Attention-Guided Interaction (Intermediate/Late Attention): SNUNet-CD [5], HANet [18], and CGNet [19].
- Category C—Token-Based, Transformer, and Exchange Interaction: BIT [6], ChangeFormer [7], and ChangerEx, the evaluated exchange-based Open-CD implementation of Changer [8].

### 4.2. Unified Training and Evaluation Protocol

All experiments followed a unified and reproducible protocol using the official Open-CD pipeline and configurations, ensuring that the observed differences primarily reflected temporal interaction design rather than inconsistent training settings.

#### 4.2.1. Runtime Environment and Unified Training Configuration

All experiments were conducted using the Open-CD framework. The runtime environment comprised a single NVIDIA Quadro RTX 4000 GPU, PyTorch 1.13.1 with CUDA 11.7, MMEngine 0.10.4, OpenCV 4.12, and Windows 10 x64. SyncBN was converted to standard BatchNorm for single-GPU execution. Because no cross-device synchronization was involved, this conversion did not change the number of samples contributing to the normalization statistics. However, no dedicated normalization ablation was conducted; therefore, architecture-specific sensitivity to BatchNorm under the applied batch settings cannot be ruled out.

All models were trained under the same configuration for 40,000 iterations using a fixed batch size of 8 bi-temporal image pairs and an input crop size of 256 × 256. Training was performed using the AdamW optimizer with an initial learning rate of 0.001 and a weight decay of 0.05, combined with a warm-up stage followed by polynomial learning-rate decay. Standard cross-entropy loss without sigmoid activation was used. To improve robustness to visual variability, the same augmentation strategy was applied across all models, including random rotation, random cropping, random horizontal and vertical flipping, and photometric distortion.

These settings were kept fixed across the evaluated models to reduce variation arising from differences in the training environment and optimization protocol. However, because the models retain their original backbone, decoder, parameterization, and overall architectural complexity, the observed performance differences cannot be attributed exclusively to temporal interaction design. Each model–dataset configuration was represented by one complete training run. Accordingly, the reported metrics are single-run point estimates, and no across-run standard deviations, confidence intervals, or statistical significance tests could be calculated. The observed differences should therefore be interpreted as descriptive comparative patterns under the adopted protocol rather than statistically validated evidence of model superiority.

#### 4.2.2. In-Domain vs. Cross-Domain Evaluation

**Figure 2:**
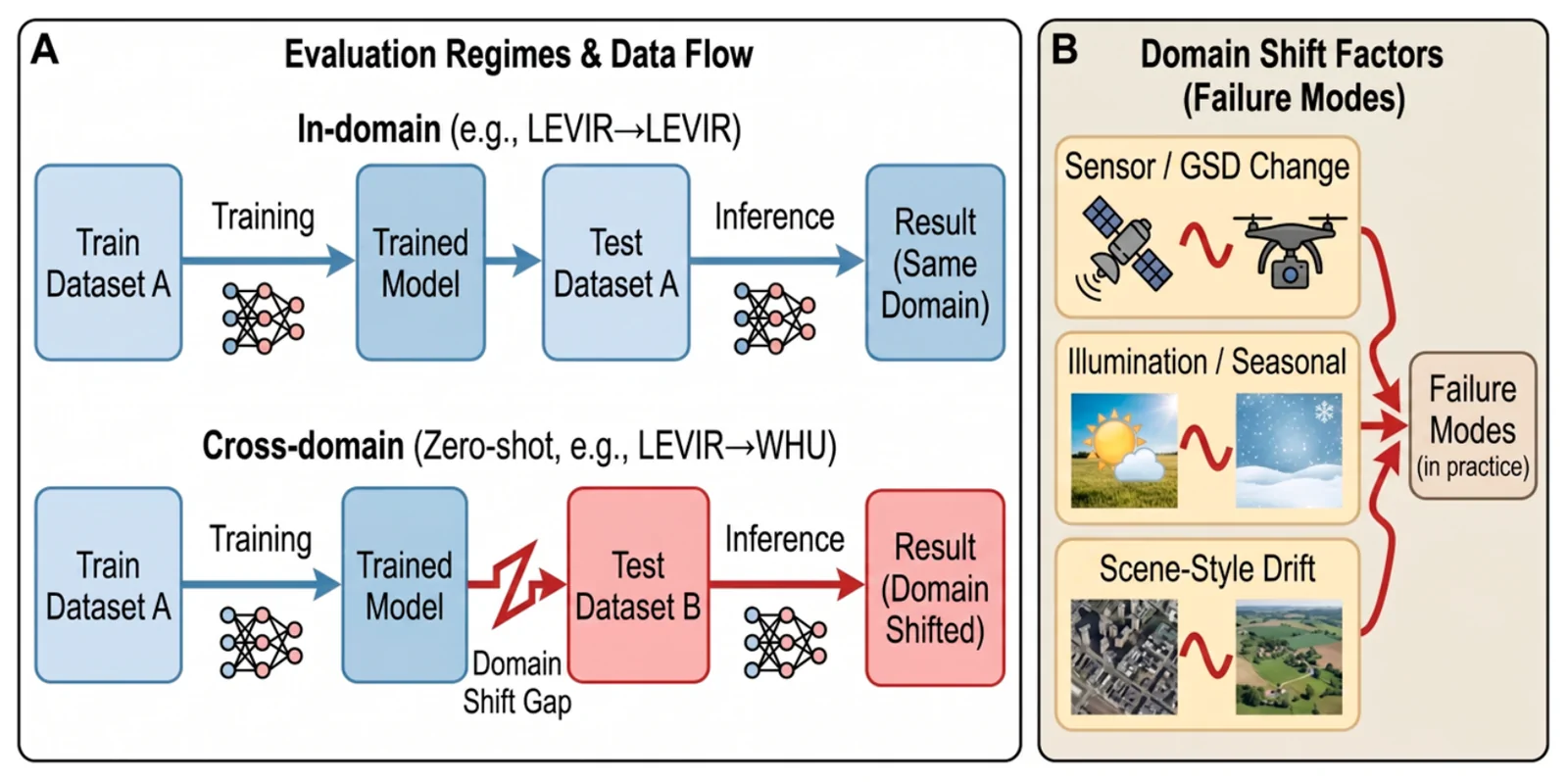
Evaluation regimes and sources of domain shift. (**A**) In-domain versus cross-domain (zero-shot) evaluation protocols. (**B**) Common sources of domain shift, including sensor/resolution differences, illumination, and scene-style drift. Blue denotes source/same-domain data, red denotes target/domain-shifted data, and arrows indicate data flow and domain-shift effects.

Two complementary protocols are adopted (Figure 2A): in-domain, where models are trained and tested on the same dataset (LEVIR→LEVIR, WHU→WHU), and cross-domain (zero-shot), where models are trained on one dataset and tested directly on the other without fine-tuning. In this study, cross-domain evaluation specifically refers to cross-dataset transfer between two optical VHR datasets, rather than cross-sensor or cross-modality transfer (Figure 2B). Relying only on in-domain evaluation can thus overestimate robustness.

#### 4.2.3. Evaluation Criteria: Emphasizing mF1 and mIoU over Accuracy

Standard pixel-level evaluation metrics are reported: overall accuracy (OA), mean Precision (mPrecision), mean Recall (mRecall), mean F1-score (mF1), and mean Intersection over Union (mIoU). The class-specific metrics are first computed separately for the changed and unchanged classes using a one-versus-rest formulation. The reported mPrecision, mRecall, mF1, and mIoU values are then obtained by macro-averaging the corresponding class-specific scores, assigning equal weight to the changed and unchanged classes.

Let C = {unchanged, changed} denote the two semantic classes. For each class c ∈ C, let ${TP}_{c}$, ${FP}_{c}$, and ${FN}_{c}$ denote the corresponding true-positive, false-positive, and false-negative pixel counts. The class-specific Precision, Recall, F1-score, and IoU are defined as follows:

(5) $${Precision}_{c}=\frac{{TP}_{c}}{{TP}_{c}+{FP}_{c}},\;c\in C$$

(6) $${Recall}_{c}=\frac{{TP}_{c}}{{TP}_{c}+{FN}_{c}},\;c\in C$$

(7) $$F_{1,c}\;=\frac{2\;{Precision}_{c}\;{Recall}_{c}}{{Precision}_{c}+{Recall}_{c}}$$

(8) $${IoU}_{c}=\frac{{TP}_{c}}{{TP}_{c}+{FP}_{c}+{FN}_{c}}$$

(9) $$\mathrm{OA}=\frac{TP+TN}{TP+TN+FP+FN}$$

The reported mean Precision and mean Recall are computed as

(10) $$\mathrm{mPrecision}=\frac{{Precision}_{changed}+{Precision}_{unchanged}}{2}$$

(11) $$\mathrm{mRecall}=\frac{{Recall}_{changed}+{Recall}_{unchanged}}{2}$$

Similarly, the reported mF1 and mIoU are computed as

(12) $$\mathrm{mF}1=\frac{F_{1,changed}+F_{1,unchanged}}{2}$$

(13) $$\mathrm{mIoU}=\frac{{IoU}_{changed}+{IoU}_{unchanged}}{2}$$

Here, TP, FP, FN, and TN in Equation (9) are defined with the changed class treated as the positive class. The macro-averaged metrics in Equations (10)–(13) follow the Open-CD/MMSegmentation IoUMetric evaluation protocol and are neither micro-averaged nor weighted by class frequency.

OA is not the primary metric here because most pixels are unchanged, so high OA can simply reflect predicting “unchanged” everywhere, an accuracy illusion that hides missed real change. This matters especially in remote sensing, where thin structures and boundaries are prone to errors from occlusion, misregistration, or shadows that barely affect OA. mF1 and mIoU are therefore emphasized as more informative indicators of class-balanced detection and region-overlap performance than OA under class imbalance [20,21,22,23]. Neither metric directly quantifies boundary accuracy.

#### 4.2.4. Robustness Evaluation Using Performance Drop and Cross-Domain Ratio

Beyond absolute in-domain and cross-domain scores, Robustness is quantified using two complementary indicators computed for a given metric (S) (typically mF1 or mIoU) between a source domain (In-domain) and a target domain (Cross-Domain):

(1) Performance Drop (ΔP): Performance Drop quantifies the relative degradation that occurs when transferring from the training domain to an unseen target domain:

(14) $$\Delta P=\frac{S_{ID}-S_{CD}}{S_{ID}}\times 100\;(\%)$$

(2) Cross-Domain (X-D) Ratio: To complement this absolute degradation measure, the X-D Ratio is computed:

(15) $$S_{X-D}=\frac{S_{CD}}{S_{ID}}$$

An X-D Ratio closer to 1 indicates stronger generalization and better retention under domain shift.

Although all metrics are reported for completeness, the analysis focuses mainly on mF1, mIoU, Performance Drop, and the X-D Ratio. While mF1 and mIoU reflect absolute detection and overlap quality, Performance Drop and X-D Ratio describe stability under cross-domain transfer. These robustness indicators are therefore interpreted jointly with absolute performance metrics, since an X-D Ratio above 1 may reflect a low in-domain baseline rather than genuine improvement.

## 5. Experimental Results

The experimental results are analyzed through the lens of temporal interaction to examine associations between interaction profiles and observed model behavior across in-domain performance, cross-dataset generalization, and characteristic failure patterns.

In-domain performance is reported first (Section 5.1), followed by cross-dataset generalization under zero-shot transfer (Section 5.2). The robustness gap between the two settings is then quantified (Section 5.3) using Performance Drop and the X-D Ratio, and complemented by a qualitative analysis (Section 5.4) of characteristic success and failure patterns across interaction categories.

### 5.1. In-Domain Performance (Training and Testing on the Same Dataset)

In-domain results (Table 2, ordered by descending mF1) show that several models with token-based or exchange interaction achieve the highest mF1 and mIoU on the two evaluated datasets. Simple-fusion baselines remain competitive in OA, particularly on LEVIR-CD, but generally show lower mIoU, indicating poorer overlap between predicted and reference regions. Result tables use Open-CD configuration names, whereas Table 1 provides the corresponding canonical publication names. These findings describe comparative in-domain performance; cross-domain robustness is examined separately in Section 5.2 and Section 5.3.

The markedly lower OA values of FC-Siam-Diff and FC-Siam-Conc on WHU-CD (88.84% and 83.55%, respectively), together with their relatively high recall values (89.59% and 86.69%), indicate extensive false-positive predictions, whereby many unchanged pixels were classified as changed. This error pattern is examined qualitatively in Section 5.4.1.

### 5.2. Cross-Dataset Generalization (Training and Testing on Different Datasets)

Cross-dataset generalization is reported in Table 3 (models ordered by descending mF1 within each transfer direction), where models trained on one dataset are evaluated directly on the other without fine-tuning, reflecting realistic deployment under domain shift.

Cross-domain performance varied substantially across models and transfer directions, confirming that strong in-domain performance alone is an insufficient predictor of robustness. ChangeFormer, BIT, and ChangerEx consistently achieved strong cross-domain mF1 and mIoU scores in both directions. However, this pattern did not extend uniformly to all interaction-rich models: under WHU→LEVIR transfer, HANet and SNUNet-CD performed below FC-Siam-Diff and FC-Siam-Conc. The results should therefore be interpreted as model- and direction-dependent trends rather than universal category-level behavior.

Furthermore, a directional transfer asymmetry is evident for most interaction-rich models: LEVIR→WHU retains higher performance than WHU→LEVIR, whereas the Category A models exhibit the reverse pattern. This asymmetry may be influenced by differences in training-set size, geographical sampling structure, and scene composition. LEVIR-CD provides a larger training set and covers 20 geographically separate regions, whereas the WHU-CD data used in this study were derived from a single geographically contiguous bi-temporal scene. Because these factors were not independently controlled, their individual contributions cannot be isolated, and the asymmetry cannot be attributed conclusively to scene diversity or dataset size alone.

### 5.3. Robustness Gap Between In-Domain and Cross-Domain Performance

**Figure 3:**
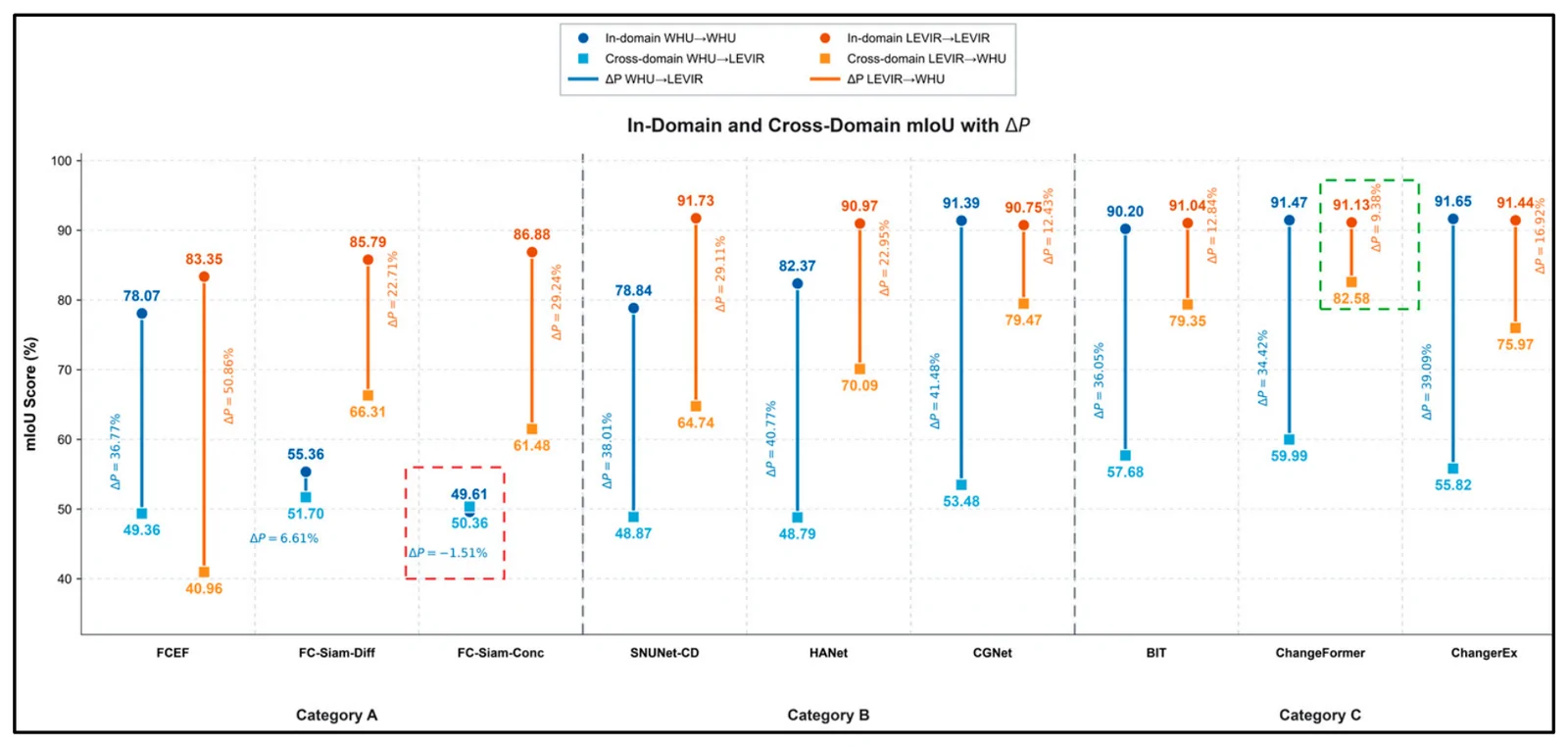
In-domain and zero-shot cross-domain mIoU across the evaluated models, grouped according to the Temporal Interaction Taxonomy (Categories A–C). Circles and squares denote in-domain and cross-domain results, respectively, while connecting lines are annotated with the signed relative performance change (ΔP); positive values indicate degradation and negative values indicate improvement. The red and green dashed boxes identify the contrasting cases discussed in Section 5.3. X-D ratios are reported in Table 4.

The robustness gap was assessed using the relative performance drop (ΔmF1/ΔmIoU) and X-D ratio defined in Section 4.2.4, with complete results reported in Table 4. For clarity, Figure 3 shows the in-domain and cross-domain mIoU scores together with the signed relative performance change (ΔP): positive values indicate degradation, whereas negative values indicate improvement. The corresponding X-D ratios remain reported in Table 4.

Under LEVIR→WHU transfer, BIT and ChangeFormer achieved the highest X-D ratios in the benchmark (0.92/0.87 and 0.94/0.91 for mF1 and mIoU, respectively). Under WHU→LEVIR, their ratios decreased to 0.69/0.64 and 0.72/0.66. FC-Siam-Conc and FC-Siam-Diff showed higher relative retention in this direction, but from substantially weaker in-domain baselines and with lower absolute cross-domain scores. Relative retention therefore reflects both the baseline and transfer performance and should not be interpreted as a universal category-level measure of robustness.

Figure 3 highlights why relative change must be interpreted alongside absolute mIoU scores. ChangeFormer (green) shows a modest relative degradation (ΔmIoU = 9.38%) while retaining strong performance (91.13% → 82.58%). In contrast, FC-Siam-Conc (red) yields a negative ΔmIoU (−1.51%) because its low in-domain score (49.61%) is slightly exceeded cross-domain (50.36%). Thus, relative change alone can be misleading without absolute context; the corresponding X-D ratios are reported in Table 4.

Transfer asymmetry was model-dependent. Six Category B and C models—SNUNet-CD, HANet, CGNet, BIT, ChangeFormer, and ChangerEx—showed smaller performance drops under LEVIR→WHU than under WHU→LEVIR (e.g., CGNet: 12.43% vs. 41.48%). In contrast, the Category A models showed the reverse pattern. Although dataset size and geographical sampling may contribute to this asymmetry, these factors and model interaction design were not independently controlled; therefore, their individual effects cannot be isolated.

Overall, robustness under the evaluated domain shifts varied with both model interaction profile and transfer direction and was not consistently predicted by architectural family or in-domain performance alone. These findings support the inclusion of cross-domain evaluation alongside conventional in-domain assessment in RSCD studies.

### 5.4. Qualitative Analysis of Temporal Interaction Behavior

This section examines how different interaction mechanisms shape change detection maps under both in-domain and cross-domain settings, providing a visual counterpart to the abstract numerical metrics and exposing characteristic error patterns.

#### 5.4.1. In-Domain Behavior: Spatial Patterns Beyond Aggregate Metrics

**Figure 4:**
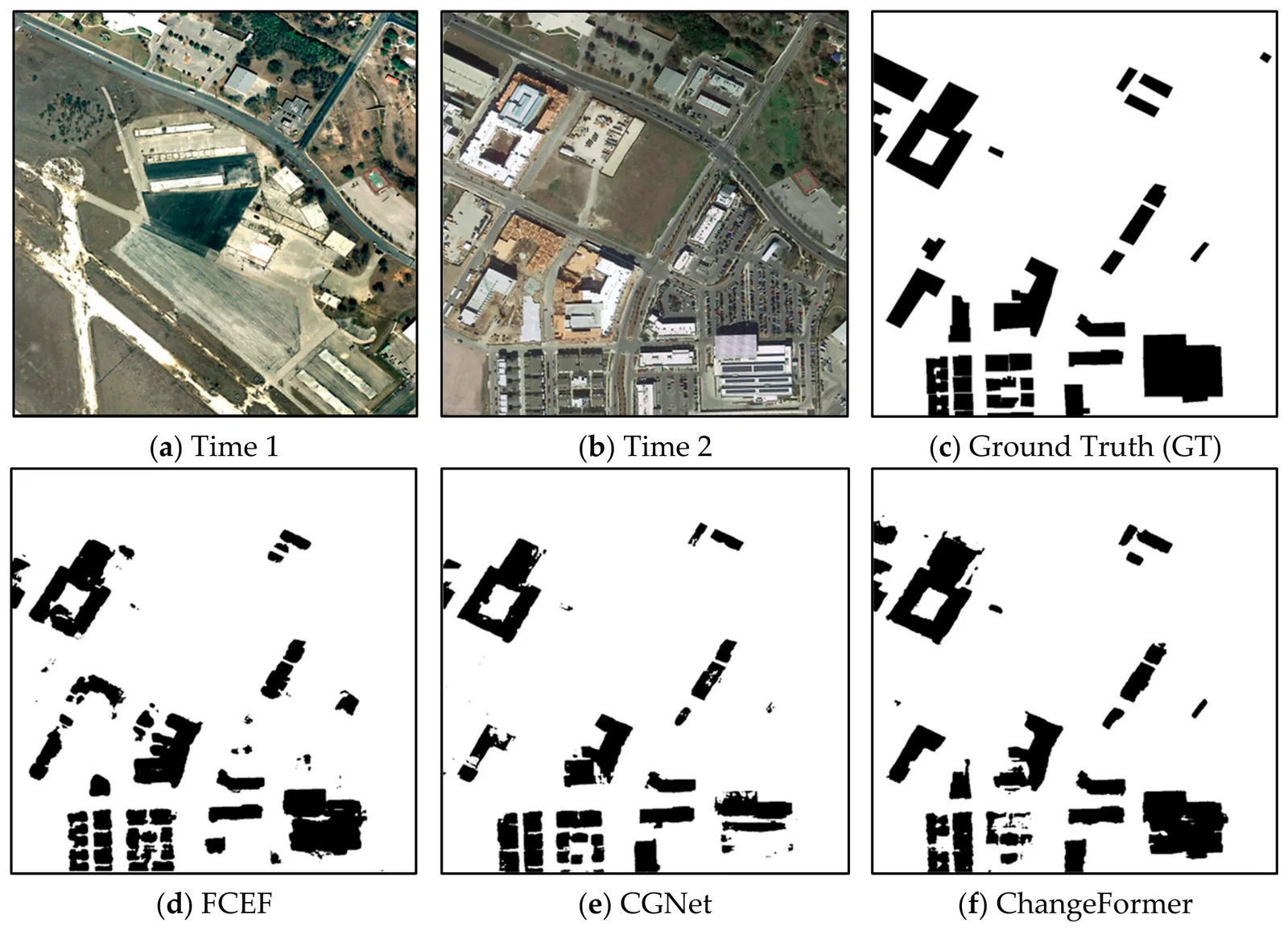
In-domain change-detection predictions on LEVIR-CD for FCEF, CGNet, and ChangeFormer, illustrating qualitative differences in spatial coherence and edge fragmentation among the selected models. Changed regions are shown in black and unchanged areas in white.

**Figure 5:**
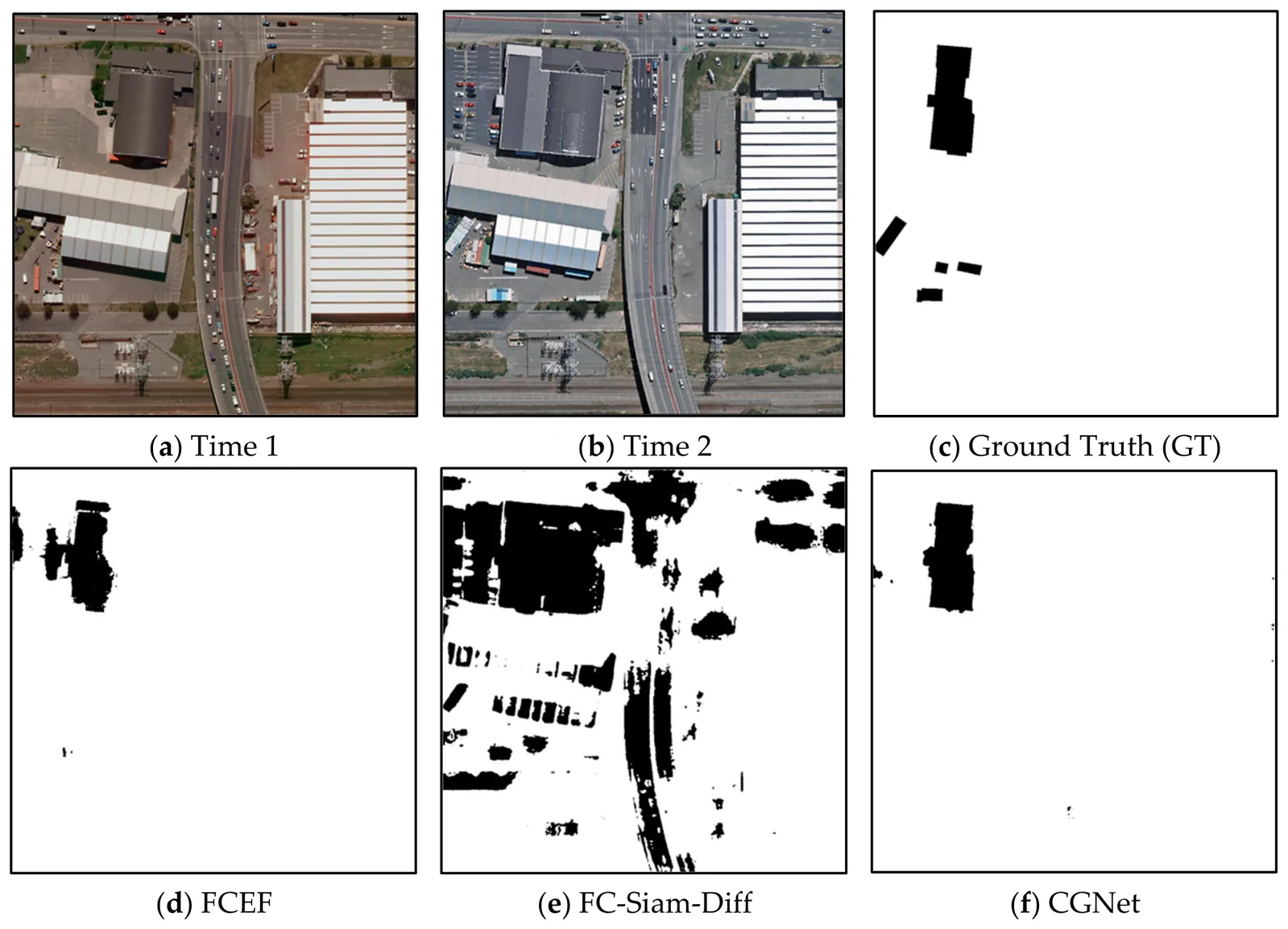
In-domain change-detection predictions on WHU-CD for FCEF, FC-Siam-Diff, and CGNet, illustrating qualitative differences in false-positive patterns, fragmentation, and spatial coherence among the selected models. Changed regions are shown in black and unchanged areas in white.

Figure 4 and Figure 5 illustrate selected in-domain predictions on LEVIR-CD and WHU-CD, respectively. In the LEVIR-CD example shown in Figure 4, qualitative differences in edge fragmentation and spatial coherence are visible among the selected models. FCEF produces more fragmented edges, CGNet shows improved structural definition, and ChangeFormer produces visually more continuous change regions. These example-specific observations are qualitatively consistent with the region-overlap scores reported in Table 2. Because no contour-sensitive metric was computed, they should not be interpreted as quantitative evidence of boundary accuracy or generalized beyond the illustrated examples.

Conversely, in the selected WHU-CD example shown in Figure 5, FCEF captures much of the changed region but also produces scattered false positives and visually irregular edges. FC-Siam-Diff shows more extensive appearance-related false detections, whereas CGNet shows fewer scattered predictions in this example. These qualitative observations illustrate that similar aggregate scores may accompany different spatial error patterns. They remain descriptive, however, because neither boundary accuracy nor the prevalence of these patterns across the full test set was quantified separately.

#### 5.4.2. Cross-Domain Qualitative Analysis of Robustness

**Figure 6:**
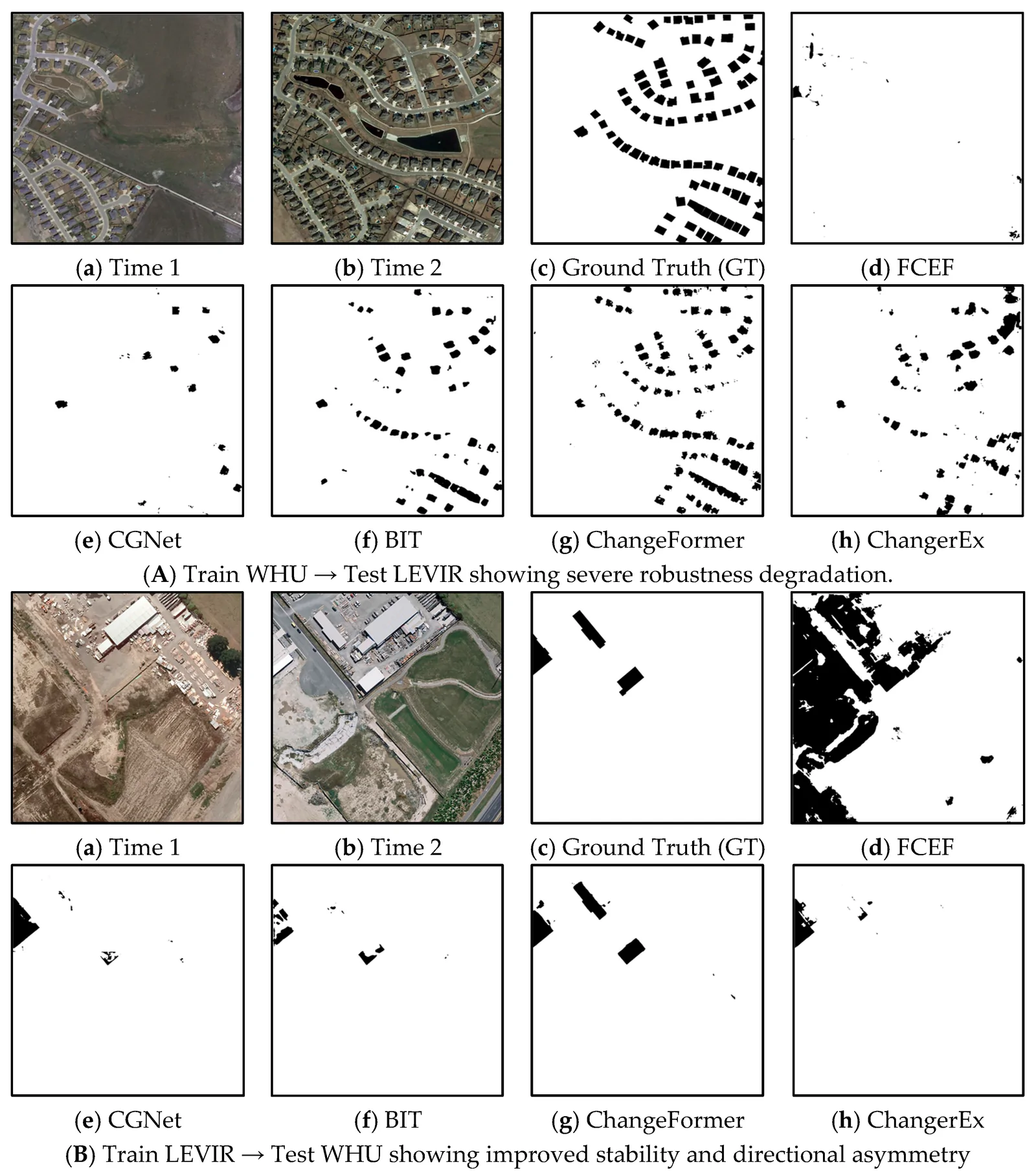
Cross-domain qualitative comparison under zero-shot transfer.

Figure 6 presents zero-shot cross-domain prediction maps, providing a visual counterpart to the quantitative trends in ΔmIoU visualized in Figure 3 and the X-D ratios reported in Table 4.

Under WHU→LEVIR transfer, the prediction maps show model-dependent differences in false positives, fragmentation, and spatial coherence. Some token-based or exchange models appear more coherent and less affected by appearance-related noise than some early- or single-stage fusion models. However, these patterns are qualitative and not uniform across models; because boundary fidelity and individual error types were not separately quantified, they should be interpreted as illustrative evidence complementing, rather than confirming, the quantitative metrics.

Under LEVIR→WHU transfer, the selected maps again show model-dependent differences in fragmentation, spatial coherence, and false-positive responses. Some models with structured interaction retain comparatively coherent change regions, whereas others produce scattered false activations. These examples broadly complement the directional patterns reported in Section 5.3 but do not establish uniform superiority for any TIT category. The quantitative results likewise show that robustness depends on both the individual model and transfer direction.

#### 5.4.3. Synthesis: The Role of Interaction Design in Robustness Pattern

These qualitative observations complement the quantitative results by showing that models with different interaction profiles exhibit distinct patterns of spatial coherence, false-positive responses, and sensitivity to appearance variation. Collectively, the observed patterns support the study’s interaction-centric hypothesis that temporal interaction design is a relevant factor in interpreting cross-domain robustness. However, because the compared models also differ in other architectural components, the present analysis does not establish temporal interaction as an independent causal or statistical predictor. These associations are examined further in the Discussion.

## 6. Discussion: Understanding Robustness Through Interaction Design

### 6.1. Associations Between Interaction Design and Generalization

The results indicate associations between temporal interaction profiles, region-overlap performance, and cross-domain robustness. The prediction maps also show differences in spatial coherence, although boundary accuracy was not quantified separately. Under the evaluated protocol, BIT, ChangeFormer, and ChangerEx achieved stronger absolute cross-domain performance than the evaluated early- or single-stage fusion models. However, this pattern was not universal: relative retention varied by model and transfer direction, and the smaller mIoU decreases of FC-Siam-Conc and FC-Siam-Diff under WHU→LEVIR partly reflected their weak in-domain baselines. TIT helps interpret these patterns through interaction timing, direction, and operator. Nevertheless, the models also differ in backbone, decoder design, parameter count, feature resolution, and complexity. Because these factors were not independently controlled, the findings represent comparative associations that motivate controlled ablations, not evidence that temporal interaction independently determines robustness or that backbone complexity is unimportant.

### 6.2. Impact of Dataset Characteristics on Transfer Asymmetry

Transfer asymmetry was model-dependent rather than universal. Most Category B and C models showed larger relative performance drops under WHU→LEVIR than under LEVIR→WHU, whereas Category A models showed the reverse pattern. This asymmetry may partly reflect dataset differences: LEVIR-CD contains more training pairs (445 vs. 226) and covers 20 geographically separate regions, whereas the WHU-CD pairs were derived from a single contiguous scene. However, scene diversity was not quantified, and training-set size, geographical coverage, scene composition, annotation characteristics, and interaction design were not independently controlled. Their individual contributions therefore cannot be isolated. The reverse pattern among Category A models further suggests that transfer behavior reflects an interaction between dataset characteristics and model design. Controlled experiments using matched training-set sizes and quantitative diversity measures are needed to separate these effects. These findings emphasize the importance of evaluating both transfer directions rather than relying on a single source–target configuration.

### 6.3. Computational Efficiency Analysis

Inference speed remains an important practical consideration for deployment. The evaluated simple-fusion models in Category A were among the fastest, requiring approximately 0.18–0.21 s/image. However, their cross-domain behavior varied by model, transfer direction, and whether robustness was assessed using absolute performance or relative retention. The evaluated attention-guided models in Category B generally required longer inference times, without showing a uniform robustness advantage across all configurations.

Several token-based or exchange models in Category C combined relatively low inference times with strong absolute cross-domain performance under the evaluated settings. This finding suggests a potentially favorable accuracy–efficiency trade-off for some Category C models when deployment conditions involve same-modality dataset shifts comparable to those examined here. Nevertheless, inference time is hardware- and implementation-dependent, and the present measurements should not be interpreted as a general computational ranking beyond the reported experimental environment.

### 6.4. Limitations and Future Work

Our findings are limited to building change detection on LEVIR-CD and WHU-CD and should not be generalized to other RSCD tasks without independent validation. Although TIT may also be relevant to applications such as land-cover change, deforestation monitoring, and disaster damage assessment, this remains untested and requires evaluation on task-specific datasets. In addition, the use of standardized Open-CD implementations may not capture custom architectural variants or proprietary optimizations that could affect the observed performance.

Although TIT is formulated as an architecture-independent analytical framework, its empirical evaluation in this study is limited to zero-shot cross-dataset transfer between LEVIR-CD and WHU-CD, both of which are optical VHR datasets. Therefore, the reported findings characterize same-modality dataset shift and should not be interpreted as evidence of cross-sensor or cross-modality generalization.

Future work will evaluate the applicability of TIT under more diverse shifts, including multi-temporal and cross-regional scenarios. Developing lightweight, hardware-efficient interaction mechanisms suitable for edge or real-time deployment would also be valuable, potentially in combination with domain-adaptation techniques such as adversarial training or test-time adaptation. Finally, systematically investigating the coupling effects between temporal fusion operators and backbone architectures could provide deeper insights into optimal interaction designs for specific deployment contexts.

A further limitation is that each model–dataset configuration was evaluated using a single complete training run. Consequently, the study does not quantify variability arising from random initialization, data ordering, or stochastic optimization, and the observed differences were not subjected to inferential significance testing. Future work should repeat each configuration across multiple predefined seeds and report measures of dispersion, confidence intervals, and appropriately corrected statistical comparisons.

Finally, mIoU quantifies region overlap rather than contour agreement. Although the qualitative prediction maps show differences in edge fragmentation and spatial coherence, boundary accuracy was not evaluated using a dedicated contour-sensitive metric. Future studies should complement region-based measures with Boundary F1, Boundary IoU, or related metrics under a predefined boundary-matching tolerance.

### 6.5. Practical Implications and Deployment Considerations

Temporal interaction design may inform model selection under dataset variation. In the evaluated optical VHR building change-detection setting, several models using structured, multi-level, token-based, or exchange interaction achieved strong absolute cross-domain performance. However, interaction design should be considered alongside backbone architecture, computational cost, absolute performance, and the anticipated transfer direction. Model evaluation should therefore combine in-domain and cross-domain scores with relative mIoU drops and X-D retention ratios, as high relative retention can be misleading when the in-domain baseline is weak.

For practitioners, this translates into the following actionable guidelines:

- When Spatial Error Patterns Are Deployment-Critical: Model selection should incorporate qualitative inspection of false-positive patterns, fragmentation, and spatial coherence in addition to aggregate region-based metrics. Where boundary performance is a deployment requirement, contour-sensitive measures such as Boundary F1 or Boundary IoU should be computed before selecting a model, because the present study did not quantify edge precision directly.
- Under Anticipated Same-Modality Dataset Shifts: Within optical VHR building change-detection settings comparable to those evaluated here, several token-based and exchange-style models achieved strong absolute cross-dataset performance. These interaction profiles may therefore be considered alongside the anticipated transfer direction, in-domain performance, and other architectural and computational requirements. Their effectiveness under cross-sensor or cross-modality shifts remains to be validated.
- For Resource-Constrained Environments: The evaluated simple-fusion models were among the fastest and may provide useful efficiency-oriented baselines when computational constraints are dominant. However, their suitability should be determined using both absolute cross-dataset performance and relative retention under the anticipated source–target relationship, because robustness varied by model and transfer direction.

When evaluating novel architectures, researchers should report both in-domain and cross-dataset metrics, together with qualitative analysis of spatial error patterns. Where boundary behavior is a central claim, this analysis should be supplemented with dedicated contour-sensitive metrics. Such interaction-aware evaluation can provide a more complete assessment of model behavior than controlled in-domain benchmarking alone, while real-world reliability requires additional validation across sensors, regions, change types, and acquisition conditions.

## 7. Conclusions

This study introduces TIT as a systematic framework for analyzing cross-dataset robustness in RSCD. It shifts attention from overall architectural complexity to the timing, direction, and operator of temporal feature interaction, according to which the evaluated networks are categorized and compared. The zero-shot cross-dataset evaluations across the LEVIR-CD and WHU-CD benchmarks yielded three main observations:

- First, temporal interaction profiles were associated with differences in region-overlap performance and cross-dataset robustness. The prediction maps also showed model-dependent differences in false detections and spatial coherence, although these error patterns and boundary fidelity were not quantified separately. Under the evaluated protocol, BIT, ChangeFormer, and ChangerEx achieved strong absolute cross-dataset performance relative to the early- or single-stage fusion baselines. However, this pattern was not consistent across all models or transfer directions, and the contribution of temporal interaction cannot be separated from differences in backbone, decoder design, parameter count, feature resolution, and model complexity.
- Second, competitive in-domain scores could mask substantial degradation under distribution shift. Therefore, relative indicators such as the X-D retention ratio should be interpreted jointly with absolute in-domain and cross-dataset metrics to avoid normalization artifacts arising from comparatively weak in-domain baselines.
- Third, cross-dataset transfer exhibited pronounced model-dependent directional asymmetry that may be associated with differences in source training-set size, geographical sampling structure, and scene composition. Because these factors were not independently controlled, their individual contributions cannot be determined from the present experiments.

The findings are limited to binary building change detection on LEVIR-CD and WHU-CD and should not be generalized to other RSCD applications without independent validation. Although TIT is not conceptually restricted to buildings, its applicability across other change types, sensors, regions, and acquisition conditions requires task-specific evaluation.

Overall, temporal interaction provides a useful analytical dimension for evaluating cross-dataset robustness among the examined models and can complement in-domain benchmarking in comparable building change-detection settings. Controlled ablations, repeated-seed experiments, and evaluations across additional change types and acquisition conditions are needed to isolate its contribution and assess the generalizability of the observed patterns.

## Figures and Tables

**Table 1:** Temporal Interaction-Based Grouping of RSCD Models.

Category	Model	Interaction Timing	Interaction Direction	Interaction Operator
Simple Fusion Baselines (Arithmetic/Aggregation)	FCEF	Early (input-level)	Symmetric	Aggregation (⊕)
	FC-Siam-Conc	Late (feature-level)	Symmetric	Aggregation (⊕)
	FC-Siam-Diff	Late (feature-level)	Symmetric	Arithmetic (Δ)
Attention- Guided Interaction (Intermediate/ Late Attention)	SNUNet-CD	Multi-level /Iterative	Bidirectional/Exchange-like	Attention + Aggregation (⊕)
	HANet	Intermediate	Directional	Attention
	CGNet	Intermediate/Late	Directional	Attention
Token/Transformer /Exchange Interaction	BIT	Intermediate /Multi-level	Bidirectional	Token-based (T) + Attention
	ChangeFormer	Multi-level	Bidirectional	Attention/ Token-based (T)
	ChangerEx	Multi-level /Iterative	Exchange (bidirectional)	Exchange (⇄)

**Table 2:** In-Domain Performance on WHU-CD and LEVIR-CD Using Open-CD Implementations.

Dataset	Model	mF1	mIoU	mPrecision	mRecall	OA	T(s/Image)
**WHU-CD** **(Train WHU → Test WHU)**	changer_ex_r18_256 × 256	**95.48**	**91.65**	96.20	94.78	99.39	0.2039
	changeformer_mit-b0_256 × 256	95.37	91.47	97.93	93.09	99.40	0.2376
	cgnet_256 × 256	95.32	91.39	95.63	95.02	99.37	1.5145
	bit_r18_256 × 256	94.61	90.20	95.74	93.54	99.28	0.2393
	hanet_256 × 256	89.46	82.37	91.54	87.59	98.63	3.4812
	snunet_c32_256 × 256	86.98	78.84	85.60	88.41	97.96	0.3722
	fcef_256 × 256	86.28	78.07	82.40	91.38	97.87	0.1816
	fc_siam_diff_256 × 256	65.14	55.36	61.20	89.59	88.84	0.1891
	fc_siam_conc_256 × 256	59.31	49.61	58.04	86.69	83.55	0.2062
**LEVIR-CD** **(Train LEVIR → Test LEVIR)**	changer_ex_r18_256 × 256	**95.36**	**91.44**	96.24	94.52	99.12	0.2238
	snunet_c32_256 × 256	95.29	91.32	95.77	94.83	99.10	0.6579
	changeformer_mit-b0_256 × 256	95.18	91.13	96.01	94.38	99.08	0.2529
	bit_r18_256 × 256	95.13	91.04	95.89	94.39	99.07	0.2684
	hanet_256 × 256	95.08	90.97	96.26	93.98	99.07	3.3852
	cgnet_256 × 256	94.96	90.75	95.98	93.98	99.05	1.7740
	fc_siam_conc_256 × 256	92.56	86.88	93.73	91.46	98.60	0.5951
	fc_siam_diff_256 × 256	91.85	85.79	93.27	90.53	98.47	0.5749
	fcef_256 × 256	90.22	83.35	90.07	90.37	98.10	0.1980

Note: Bold values indicate the highest mF1 and mIoU within each dataset.

**Table 3:** Cross-Dataset Generalization Performance under Zero-Shot Transfer (WHU→LEVIR and LEVIR→WHU) Using Open-CD Framework.

Transfer	Model	mF1	mIoU	mPrecision	mRecall	OA	T(s/Image)
**WHU → LEVIR** **(Train WHU → Test LEVIR)**	changeformer_mit-b0_256 × 256	**68.33**	**59.99**	93.08	62.26	96.02	0.2536
	bit_r18_256 × 256	65.34	57.68	89.38	60.14	95.73	0.2396
	changer_ex_r18_256 × 256	62.79	55.82	84.09	58.48	95.45	0.2071
	cgnet_256 × 256	59.20	53.48	89.27	55.87	95.39	1.5245
	fc_siam_diff_256 × 256	56.50	51.70	74.35	54.35	94.96	0.2065
	fc_siam_conc_256 × 256	54.16	50.36	75.25	52.89	94.96	0.2058
	fcef_256 × 256	54.01	49.36	54.90	53.50	92.36	0.1904
	snunet_c32_256 × 256	52.25	48.87	55.24	51.82	93.72	0.5613
	hanet_256 × 256	51.57	48.79	59.23	51.41	94.51	3.3742
**LEVIR → WHU** **(Train LEVIR → Test WHU)**	changeformer_mit-b0_256 × 256	**89.61**	**82.58**	91.52	87.88	98.65	0.2529
	cgnet_256 × 256	87.31	79.47	91.16	84.16	98.42	1.7797
	bit_r18_256 × 256	87.21	79.35	93.10	82.82	98.46	0.2428
	changer_ex_r18_256 × 256	84.52	75.97	89.93	80.48	98.14	0.1845
	hanet_256 × 256	79.49	70.09	75.61	85.00	96.69	3.5324
	fc_siam_diff_256 × 256	76.07	66.31	69.98	89.78	95.09	0.1851
	snunet_c32_256 × 256	74.49	64.74	68.66	88.17	94.66	0.5714
	fc_siam_conc_256 × 256	71.34	61.48	65.60	90.05	92.92	0.2002
	fcef_256 × 256	51.15	40.96	55.07	81.54	72.37	0.1881

Note: Bold values indicate the highest mF1 and mIoU within each transfer direction.

**Table 4:** Relative Performance Drop from In-Domain to Cross-Domain Evaluation.

Category	Model	Domain Transfer Direction	Performance Drop (%) ↓		X-D Ratio	
			ΔmF1	ΔmIoU	mF1	mIoU
A	FCEF	WHU→LEVIR	37.40	36.77	0.63	0.63
		LEVIR→WHU	43.31	50.86	0.57	0.49
	FC-Siam-Conc	WHU→LEVIR	8.68	−1.51	0.91	1.02
		LEVIR→WHU	22.93	29.24	0.77	0.71
	FC-Siam-Diff	WHU→LEVIR	13.26	6.61	0.87	0.93
		LEVIR→WHU	17.18	22.71	0.83	0.77
B	SNUNet-CD	WHU→LEVIR	39.93	38.01	0.60	0.62
		LEVIR→WHU	21.83	29.11	0.78	0.71
	HANet	WHU→LEVIR	42.35	40.77	0.58	0.59
		LEVIR→WHU	16.40	22.95	0.84	0.77
	CGNet	WHU→LEVIR	37.89	41.48	0.62	0.59
		LEVIR→WHU	8.06	12.43	0.92	0.88
C	BIT	WHU→LEVIR	30.94	36.05	0.69	0.64
		LEVIR→WHU	8.33	12.84	0.92	0.87
	ChangeFormer	WHU→LEVIR	28.35	34.42	0.72	0.66
		LEVIR→WHU	**5.85**	**9.38**	**0.94**	**0.91**
	ChangerEx	WHU→LEVIR	34.24	39.09	0.66	0.61
		LEVIR→WHU	11.37	16.92	0.89	0.83

Note: Bold values indicate the lowest performance drop and highest X-D ratio for the LEVIR→WHU transfer direction.

## Data Availability

The datasets used in this study are publicly available. The LEVIR-CD and WHU-CD datasets can be accessed through their original sources, as cited in References [10] and [11], respectively.

## References

[1] Khelifi L., Mignotte M. (2020). Deep Learning for Change Detection in Remote Sensing Images: Comprehensive Review and Meta-Analysis. IEEE Access.

[2] Corley I., Robinson C., Ortiz A. (2024). A Change Detection Reality Check. arXiv.

[3] Cheng G., Huang Y., Li X., Lyu S., Xu Z., Zhao H., Zhao Q., Xiang S. (2024). Change Detection Methods for Remote Sensing in the Last Decade: A Comprehensive Review. Remote Sens..

[4] Daudt R.C., Le Saux B., Boulch A. Fully Convolutional Siamese Networks for Change Detection. Proceedings of the 2018 25th IEEE International Conference on Image Processing (ICIP).

[5] Fang S., Li K., Shao J., Li Z. (2022). SNUNet-CD: A Densely Connected Siamese Network for Change Detection of VHR Images. IEEE Geosci. Remote Sens. Lett..

[6] Chen H., Qi Z., Shi Z. (2021). Remote Sensing Image Change Detection with Transformers. IEEE Trans. Geosci. Remote Sens..

[7] Bandara W.G.C., Patel V.M. (2022). A Transformer-Based Siamese Network for Change Detection. Proceedings of the IGARSS 2022—2022 IEEE International Geoscience and Remote Sensing Symposium.

[8] Fang S., Li K., Li Z. (2023). Changer: Feature Interaction Is What You Need for Change Detection. IEEE Trans. Geosci. Remote Sens..

[9] Li K., Jiang J., Han C., Codegoni A., Deng Y., Chen K., Zheng Z., Chen H., Liu Z., Gu Y. Open-CD: A Comprehensive Toolbox for Change Detection. Proceedings of the 33rd ACM International Conference on Multimedia (MM ’25).

[10] Chen H., Shi Z. (2020). A Spatial-Temporal Attention-Based Method and a New Dataset for Remote Sensing Image Change Detection. Remote Sens..

[11] Liu Y., Pang C., Zhan Z., Zhang X., Yang X. (2021). Building Change Detection for Remote Sensing Images Using a Dual-Task Constrained Deep Siamese Convolutional Network Model. IEEE Geosci. Remote Sens. Lett..

[12] Ronneberger O., Fischer P., Brox T., Navab N., Hornegger J., Wells W.M., Frangi A.F. (2015). U-Net: Convolutional Networks for Biomedical Image Segmentation. Medical Image Computing and Computer-Assisted Intervention—MICCAI 2015.

[13] Huang Y., Zhang P. (2024). CSDACD: Domain-Adaptive Change Detection Network for Cross-Seasonal Remote Sensing Images. IEEE Geosci. Remote Sens. Lett..

[14] Fan R., Xie J., Yang J., Hong Z., Xu Y., Hou H. (2024). Multiscale Change Detection Domain Adaptation Model Based on Illumination-Reflection Decoupling. Remote Sens..

[15] Liu Q., Ren K., Meng X., Shao F. (2023). Domain Adaptive Cross Reconstruction for Change Detection of Heterogeneous Remote Sensing Images via a Feedback Guidance Mechanism. IEEE Trans. Geosci. Remote Sens..

[16] Wang D., Shelhamer E., Liu S., Olshausen B., Darrell T. Tent: Fully Test-Time Adaptation by Entropy Minimization. Proceedings of the International Conference on Learning Representations (ICLR).

[17] Sinha S., Gehler P., Locatello F., Schiele B. TeST: Test-Time Self-Training under Distribution Shift. Proceedings of the IEEE/CVF Winter Conference on Applications of Computer Vision (WACV).

[18] Han C., Wu C., Guo H., Hu M., Chen H. (2023). HANet: A Hierarchical Attention Network for Change Detection with Bitemporal Very-High-Resolution Remote Sensing Images. IEEE J. Sel. Top. Appl. Earth Obs. Remote Sens..

[19] Han C., Wu C., Guo H., Hu M., Li J., Chen H. (2023). Change Guiding Network: Incorporating Change Prior to Guide Change Detection in Remote Sensing Imagery. IEEE J. Sel. Top. Appl. Earth Obs. Remote Sens..

[20] Sokolova M., Lapalme G. (2009). A Systematic Analysis of Performance Measures for Classification Tasks. Inf. Process. Manag..

[21] Peng D., Liu X., Zhang Y., Guan H., Li Y., Bruzzone L. (2025). Deep Learning Change Detection Techniques for Optical Remote Sensing Imagery: Status, Perspectives and Challenges. Int. J. Appl. Earth Obs. Geoinf..

[22] Ma L., Liu Y., Zhang X., Ye Y., Yin G., Johnson B.A. (2019). Deep Learning in Remote Sensing Applications: A Meta-Analysis and Review. ISPRS J. Photogramm. Remote Sens..

[23] Tang H., Wang H., Zhang X. (2022). Multi-Class Change Detection of Remote Sensing Images Based on Class Rebalancing. Int. J. Digit. Earth.

